# The Role of miRNAs in the Pathophysiology of Liver Diseases and Toxicity

**DOI:** 10.3390/ijms19010261

**Published:** 2018-01-16

**Authors:** Florian Schueller, Sanchari Roy, Mihael Vucur, Christian Trautwein, Tom Luedde, Christoph Roderburg

**Affiliations:** 1 Department of Medicine III, University Hospital RWTH Aachen, Pauwelsstrasse 30, 52074 Aachen, Germany; fschueller@ukaachen.de (F.S.); sroy@ukaachen.de (S.R.); mvucur@ukaachen.de (M.V.); ctrautwein@ukaachen.de (C.T.); tluedde@ukaachen.de (T.L.); 2 Division of Gastroenterology, Hepatology and Hepatobiliary Oncology, University Hospital RWTH Aachen, Pauwelsstrasse 30, 52074 Aachen, Germany

**Keywords:** liver, miRNAs, acute liver injury, liver toxicity, liver cirrhosis

## Abstract

Both acute and chronic liver toxicity represents a major global health burden and an important cause of morbidity and lethality worldwide. Despite epochal progress in the treatment of hepatitis C virus infections, pharmacological treatment strategies for most liver diseases are still limited and new targets for prevention or treatment of liver disease are urgently needed. MicroRNAs (miRNAs) represent a new class of highly conserved small non-coding RNAs that are involved in the regulation of gene expression by targeting whole networks of so called “targets”. Previous studies have shown that the expression of miRNAs is specifically altered in almost all acute and chronic liver diseases. In this context, it was shown that miRNA can exert causal roles, being pro- or anti-inflammatory, as well as pro- or antifibrotic mediators or being oncogenes as well as tumor suppressor genes. Recent data suggested a potential therapeutic use of miRNAs by targeting different steps in the hepatic pathophysiology. Here, we review the function of miRNAs in the context of acute and chronic liver diseases. Furthermore, we highlight the potential role of circulating microRNAs in diagnosis of liver diseases and discuss the major challenges and drawbacks that currently prevent the use of miRNAs in clinical routine.

## 1. miRNAs

MicroRNAs (miRNAs) are small, non-protein coding, single-stranded RNAs of ~22 nucleotides length, which are transcribed by RNA polymerase II and III. MiRNAs were first described 1993 in *C. elegans* and demonstrated to be capable to regulate gene expression post-transcriptionally via binding to the 3′ or 5′ untranslated region (UTR) of their target mRNAs [1,2,3,4,5]. Hence, miRNAs can regulate diverse biological aspects, including cell proliferation, differentiation, cell death, as well as organ development and the maintenance of organ physiology [6]. Due to their broad physiological function, miRNAs were intensively studied in the last decade and more than 1800 miRNAs have been identified so far in human [7]. In silico data predicted that more than 45,000 miRNA target sites are present in human DNA and that expression of more than 60% of all protein-coding genes are regulated by miRNAs [8]. 

After transcription, the resulting 500–3000 nucleotides long pri-miRNAs are processed within the nucleus by the so-called “microprocessor complex” into precursor miRNAs (pre-miRNA, approximately 70 nucleotides long). Pre-miRNAs are released in the cytoplasm via *exportin-5*-mediated nuclear export. Within the cytoplasm pre-miRNAs are cleaved by the RNase III endonuclease “Dicer” into ~22 nucleotides long, double stranded miRNAs.

**Figure 1:**
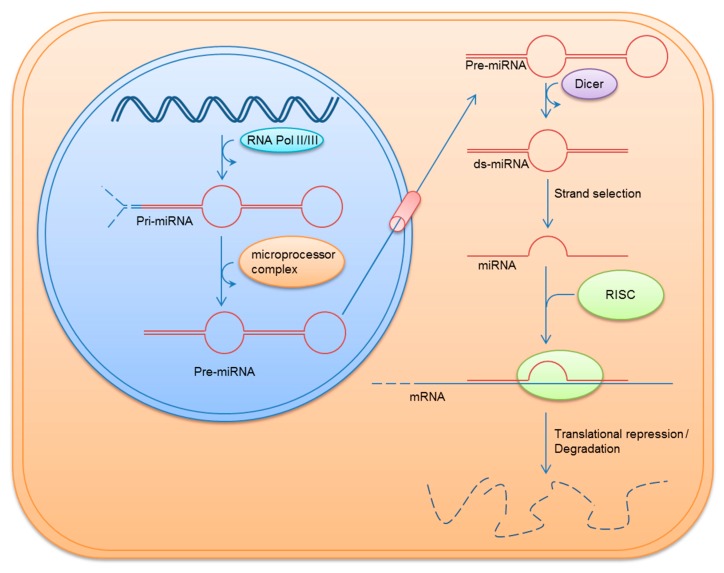
The biogenesis of MicroRNAs (miRNAs). RNA Pol, RNA polymerase; miRNA, microRNA; pri-miRNA, primary miRNA; pre-miRNA, precursor miRNA; ds-miRNA, double stranded miRNA; RISC, RNA-induced silencing complex; mRNA, messenger RNA.

After guide strand selection, a single-stranded, mature miRNA is bound to Argonaute which is part of the “RNA-induced silencing complex” (RISC). Besides, Argonaute, guide strand selection is based on different proteins like DICER, TRBP, PACT (protein activator of dsRNA-dependent protein kinase), or Xrn-1/2. However, the exact mechanisms and interaction of these proteins during guide strand selection is largely unknown, guide strands seem to have a weaker binding and an U-bias at its 5′-end as well as larger amounts of purines than passenger strands displaying a C-bias at their 5′-end and an excess of pyrimides. Therefore thermodynamic properties may play a crucial role in guide strand selection [9]. RISC can induce the posttranscriptional or translational repression of mRNAs via binding of the loaded miRNA to the 3′ or 5′ UTR of its target mRNAs (Figure 1). In contrast to complete complementary binding inducing degradation of target mRNA, partial complementary binding leads to translational repression [1,3,10,11]. 

Considering that one miRNA can have multiple mRNA targets and each target can be regulated by different miRNAs, they are capable of regulating essential cell biological processes. In contrast, deregulation of various miRNA profiles is associated with different acute and chronic liver diseases including viral hepatitis, steatohepatitis, liver fibrosis, cirrhosis, and hepatocellular carcinoma (HCC) [12]. Since miRNAs are extremely stable in body fluids, such as serum samples, they have been extensively studied in recent years in order to develop their potential as biomarkers for liver diseases. In this review, we summarize available data on the role of miRNAs in acute and chronic liver toxicity (summarized in Table 1). When considering the enormous progress in the field of miRNA research we have focussed on selected miRNAs rather than providing an enumeration of all miRNAs associated to liver diseases. Of note, many studies presented within this review rely on hypothesis-driven research from mice. Undoubtedly, animal research has provided important results that have helped to improve outcome of patients with liver diseases and represent an important source for further clinical research [13,14]. Nevertheless, despite miRNAs represent highly conserved molecules, it is important to acknowledge known limitations in clinical translation from mouse to man. Wherever available, we have tried to correlate data from rodent models to findings from patients with the respective liver diseases. 

## 2. miRNAs in the Physiology of the Liver

Since their discovery in 1993, miRNAs were studied to clarify their physiological role in organ homeostasis, tissue development, and regeneration. The most important hepatic miRNA, miR-122 comprises approximately 70% of all hepatic miRNAs. Its downregulation leads to decreased plasma cholesterol levels, increased hepatic fatty-acid oxidation, as well as to a reduction of hepatic fatty-acid and cholesterol synthesis [44]. Furthermore, miR-122 inhibition leads to an upregulation of *hemochromatosis* (Hfe), *hemojuvelin* (Hjv), *bone morphogenetic protein receptor type 1A* (Bmpr1a), and *hepcidin antimicrobial peptide* (Hamp), which control systemic iron levels [45]. However, hepatic levels of miR-122 are nearly constant, they are associated with circadian gene expression in the liver [46], because knockdown of miR-122 has resulted in dysregulation of many mRNAs, which accumulated in a circadian fashion. Furthermore, miRNA expression profiles were linked to hepatocyte proliferation and liver regeneration [47]. In vitro, *hepatocyte nuclear factor 4 alpha* (HNF4α) was identified as a target gene of miR-24 and miR-34a. Downregulation of HNF4α resulted in suppression of cytochrome P450 and a reduction of HepG2 population in S-phase [47]. Interesting insights on the role of miRNAs in liver development were provided by Hand et al. [48]. Conditional knockout of DICER1 in hepatoblast-derived cells led to a significant downregulation of miR-122, miR-192, and miR-194. However *AfpCre*;*Dicer1^flox/flox^* mutants displayed no altered phenotype directly after birth, they developed at 2–4 month of age progressive hepatocyte damage, as seen by increased AST and ALT levels, an increased liver mass accompanied by an increased proliferation and apoptosis [48]. A comprehensive review on miRNA function in liver development is given in [49]. 

In conclusion, the provided examples demonstrate the diverse role of miRNAs in maintaining liver homeostasis, and therefore highlight miRNA’s involvement in acute and chronic liver diseases.

## 3. The Role of miRNAs in Acute Liver Toxicity

Acute liver failure (ALF) is associated by a severe loss of hepatic cell function without preexisting liver injury. Various toxins like drug intoxication (e.g., acetaminophen (APAP) overdose), viral or autoimmune hepatitis, as well as Wilson’s disease or Budd-Chiari syndrome can lead to ALF [50,51]. Even though liver transplantation has increased overall survival, mortality rates of still up to 55–75% indicate that ALF remains a rare, but challenging, clinical condition [52]. The term drug induced liver injury DILI comprises both true drug induced liver injury, but also liver injury caused by non-drug products. This kind of liver injury should be classified as herb-induced liver injury (HILI). Of note, both DILI and HILI should be further subdivided e.g., into an hepatocellular or cholestatic/mixed type as well as into an idiosyncratic or an intrinsic type, depending on the specific toxic action of the offending agent. At present, no biomarker allowing for the early recognition or subclassification of DILI or HILI is established. In clinical practice, repetitive measurements of ALT or ALP in connection with causality assessment using the RUCAM score is recommended (summarized in detail in [53]). For novel, e.g., miRNA based biomarkers to be used in DILI or HILI, major issues are still to be addressed: Most importantly, the need to be (i) liver specific and (ii) drug specific, especially in patients with high causality gradings using RUCAM. As, in contrast to protein based biomarkers, miRNAs might reflect complex pathophysiological processes, it seems likely that miRNAs might fulfill these requirements better than previous biomarkers. 

A broad variety of animal models like APAP, CCl_4_, thioacetamide (TAA), concanavalin A (ConA), lipopolysaccharide (LPS), and d-Galactosamine (d-Gal) administration, as well as surgical techniques like ischemia and reperfusion (I/R) have been intensively used to study the functional mechanisms of liver intoxication [54]. Array based miRNA profiling was performed in basically all of these models, and a vaste amount on information about miRNAs deregulated in the context of acute liver toxicity is presently available. In a broad comparative microarray study, Fukushima et al. investigated alteration of various miRNAs after APAP and CCl_4_ administration in rats and found six miRNAs (miR-153, miR-302b_AS, miR-337, miR-363, miR-409-5p, miR-542-3p) to be upregulated and eight miRNAs (miR-29c_AS, miR-298, miR-327, miR-342, miR-370, miR-376c, miR-494, miR-503) to be downregulated in both models of hepatotoxicity [55]. Highly liver-specific miR-122 accounts for approximately 70% of all miRNA expressed in the liver, and was therefore intensively studied in context of liver injury [15,16,17,18,44,56]. Beside miR-122, miR-192 was found to be increased in sera of mice after APAP administration when compared to controls and elevation occurred in a dose- and exposure dependent manner [15]. In regard to that, both miRNAs were elevated earlier than serum transferase levels [15], underlining their diagnostic value in comparison to standard ALF markers. Increase of miRNA-122 serum levels were confirmed by Bala et al. describing a time-dependent elevation and correlation with ALT levels [56]. In agreement, miR-122 serum levels were also elevated in an I/R mice model, being correlated with AST/ALT levels, as well as with hepatic cell death detected by TUNEL staining [16]. When considering that miR-122 increases in the supernatant after hepatocyte injury in vitro, these data indicate that miR-122 might represent a surrogate for hepatocyte death in liver injury [16]. Interestingly, miR-122 and miR-192 elevation could be confirmed in sera of APAP induced ALF patients [17] and are consistent with data from high throughput sequencing of patients with APAP overdose [18]. In these patients, circulating levels of 36 miRNAs were increased as compared to controls [18]. Furthermore, miR-122, miR-192, miR-194, miR-483, and miR-210 were identified as liver-enriched after APAP overdose [18]. MiR-122 elevation upon liver injury was also confirmed in a cohort of ALF patients [51]. 

Since miRNAs generally have a less complex chemical structure, lacking post-processing modifications, and were shown to be extremely stable, numerous studies have been conducted in recent years to investigate the diagnostic and prognostic potential of miRNAs as a reliable biomarker for acute liver failure [15,51,57,58]. Exemplarily, an early increase in circulating miR-122 and miR-192 levels compared to serum aminotransferase [15] showed that miRNAs have the ability to improve the early detection of liver damage, and thus improve the overall survival in ALF patients. While standard serum markers, such as AST or ALT, are not entirely hepato-specific and display limited prognostic power [18,59,60], new biomarkers need to be established to increase overall survival in patients with acute liver damage. 

Recently, the suitability of miR-223 as a biomarker for acute liver injury was comprehensively studied [21]. Up to date, only few, but interesting studies are available about miR-223 expression in liver diseases [21,61,62,63]. MiR-223 was identified as a regulator of hematopoietic linage differentiation and as a regulator of diverse immune cell functions [64,65]. Yu and colleagues showed that miR-223 was upregulated in livers from mice after acute I/R induced liver injury [62]. This finding was confirmed by Schueller et al. showing that hepatic miR-223 expression in mice were consistently elevated in I/R, as well as in CCl_4_, APAP, and ConA induced acute liver injury models when compared to respective controls [21]. In a cohort of ALF patients, miR-223 hepatic expression and serum levels were elevated as compared to controls. Interestingly, elevated hepatic expression of miR-223 reflected an impaired prognosis as those patients, who did not spontaneously recover, demonstrated significantly higher hepatic miR-223 levels than patients that spontaneously recovered from ALF [21]. However, further investigations are crucially needed, these data strongly indicate the potential of miR-223 to suit as a biomarker of ALF. The functional involvement of miR-223 in acute liver injury is however still unclear. Qadir et al. impressively demonstrated that *miR-223^−/−^* mice were protected against Fas-induced hepatocyte apoptosis and liver injury [20]. In contrast, knockout of miR-223 had no impact on the severity of liver injury in mice after I/R or CCl_4_ induced acute liver injury [21]. Of note, findings of Qadir et al. were confirmed in this study. As *Fas^−/−^* mice behaved similar to wild type (wt) mice in I/R induced liver injury, this model seems to be independent from Fas-signaling [66], which might be an explanation for these contradictory results. Furthermore, deletion of miR-223 did not affect the degree of liver damage in mice upon acute ethanol gavage [61]. In summary, further knockout based studies should be conducted to unravel the precise role of miR-223 in the different aetiologies of acute liver diseases. In concordance to the observed inter-model differences in miR-223*^−/−^* mice, miR-150 deficiency in mice had a protective effect on Fas-induced liver injury, whereas mortality and liver damage of *miR-150^−/−^* was unaltered in LPS/GalN mice [22]. Interestingly, Jo2-treated *miR-150^−/−^* mice displayed higher expression of *Akt* when compared to wild type mice and inhibition of *Akt* via *Akt inhibitor V* could restore Fas-induced liver injury. Furthermore, the authors could identify *Akt1* and *Akt2* as direct target of miR-150 via luciferase reporter assays [22]. The protective effect of miR-223 and miR-150 in Fas-induced liver injury impressively show, that deregulation of miRNAs not only point out their potential use as a biomarker, but they may function as well as a therapeutic option for ALF patients. As an example, Yang et al. showed that miR-125b-5p overexpression ameliorated APAP-induced hepatic toxicity in mice [67]. ALF prevention was further linked to a reduced protein expression of *kelch-like ECH-associated protein1* (*Keap1*), which was previously shown to attenuate ALF [67,68]. In a comparable approach, Yuan and colleagues investigated the therapeutic potential of miR-155 in mice after APAP-induced liver injury [69]. MiR-155 was found to be upregulated in both liver tissue and blood samples after APAP administration. Of note, *miR-155^−/−^* mice showed increased AST and ALT levels and an increase of inflammatory mediators like TNFα or IL6. Importantly, miR-155 deficiency led to NF-κB activation via increased p65 and IKK_ε_ expression. In contrast, miR-155 agomir administration had a protective effect on APAP-induced liver damage, represented by reduced AST and ALT serum levels [69]. In a different model of toxin-based liver injury by dioxin administration miR-122 and miR-101a were downregulated. Interestingly, reduced miR-101a levels were associated with an increased expression of its target gene Cox2, which might suppress the onset of Dioxin-induced liver damage via the Cox2 selective inhibitor NS-398 [70]. In another comprehensive study, miR-192-5p levels were analyzed in different in vivo models of acute liver injury, as well as in a cohort of patients with acute liver injury [23]. MiR-192-5p expression was shown to be restricted to hepatocytes and intrahepatic expression of miR-192-5p was downregulated after I/R, as well as after CCl_4_ induced liver damage. Of note, this finding was confirmed in patients with acute liver injury. In contrast, miR-192-5p serum levels were increased after I/R and correlated with the degree of liver damage and the presence of hepatic cell death. Interestingly, functional experiments showed that the downregulation of miR-192-5p had a protective effect in HepG2 cells after H_2_O_2_ treatment, suggesting a role of miR-192-5p in limiting liver injury. *Zeb2*, an important regulator of cell death, was identified via luciferase reporter assay as a potential target gene of miR-192-5p mediating its function in vitro [23]. Similarly, the functional role of miR-1224 was analyzed in the context of acute liver damage [25]. Consistently, miR-1224 was upregulated in mice after induction of liver injury via I/R, CCl_4_, and APAP intoxication. Furthermore, upregulation of miR-1224 was associated with impaired proliferation as well as elevated apoptosis. In hepatocytes, miR-1224 function was mediated by repressing the anti-apoptotic gene *Nfib*. Interestingly, increase of miR-1224 levels was confirmed in liver and serum samples of ALF patients and was linked to an unfavorable prognosis [25].

Interesting functional insides in the role of deregulated miRNAs during liver injury were also provided by John et al. [51]. In comparison to non-recovered ALF patients, circulating levels of miR-122, miR-21, and miR-221 were increased in patients spontaneously recovered from ALF. Higher hepatocyte proliferation, and therefore higher liver tissue regeneration was associated with decreased hepatic expression of the respective miRNA target genes *heme-oxgenase-1*, *programmed cell death 4*, *p27,* and *p57* [51].

With its broad usage of herbal agents Traditional Chinese Medicine (TCM) has gained increasing attention. However, discussion has emerged in recent years whether herb induced liver injury from herbal TCM could be a major issue [71]. To provide further insights into the role of miRNAs in herbal induced liver injury, Zheng et al. identified eight dysregulated miRNA (upregulated: miR-21a-3p, miR-5099, miR-3960, 3081-5p, miR-2861, miR-680; downregulated: miR-139-5p and 199a-5a) after exposition of mice to hepatotoxic *Fructus Meliae Toosendan* water extracts [72]. In this study, the authors linked miRNA dysregulation to several cellular functions, like cell growth, proliferation, and development [72]. In another broad approach, Su et al. investigated miRNA profiles in liver tissue and serum samples of rats with *Dioscorea bulbifera* (TCM herb) induced liver injury and identified a variety of miRNAs being dysregulated. In this study, the authors presented circulating miR-122, miR-192, and miR-193 as a new panel for herb-induced liver injury diagnosis [19].

In conclusion, investigation of deregulated miRNA and their functional target genes might provide new insights in the pathophysiology of acute liver injury. Therefore, they could not only provide a source of new diagnostic and prognostic biomarkers, but might reveal new therapeutic options for liver intoxication. 

## 4. The Role of miRNAs in Chronic Alcoholic Liver Toxicity

Alcoholic liver disease (ALD) represent a major global health burden. The disease includes acute liver toxicity, alcoholic fatty liver, alcoholic cirrhosis, and finally, HCC [73,74]. In this context, the role of miRNAs in the pathophysiology of ALD has been widely studied [75,76]. Dolganiuc et al. recently demonstrated in a murine model of ethanol-induced steatohepatitis that ethanol consumption led to an up and downregulation of 1% of all analyzed miRNAs, respectively [76]. Most importantly, miRNAs have been demonstrated to play a crucial role in regulating the process of liver inflammation caused by Lipopolysaccharide (LPS) upon ethanol ingestion. Most importantly, chronic alcohol treatment resulted in a time-dependent increase of miR-155 expression in RAW 264.7 macrophages. Alcohol pretreatment augmented LPS-induced miR-155 expression in these cells [77,78]. In line, Bala et al. demonstrated that a deletion of miR-155 protects mice from alcohol-induced steatosis and inflammation [26]. Moreover, miR-155-KO mice subjected to an alcohol diet were protected from liver fibrosis according to analysis of hydroxyproline liver content and α-SMA expression, highlighting the crucial role of miR-155 in ethanolic liver toxicity [26]. Besides miR-155, deregulation of several other miRNAs was described in livers from mice fed with an ethanol-containing diet (Lieber–DeCarli diet): while expression of miR-320, miR-486, miR-705, and miR-1224 were elevated, a decreased expression was described for miR-27b, miR-214, miR-199a-3p, miR-182, miR-183, miR-200a, and miR-322 [26,76]. Similarly, expression of the proinflammatory miR-375 was shown to be increased in patients with alcohol consumption [79]. Finally, Dippold RP et al. demonstrated the deregulation of a panel of miRNAs, including miR-34a, miR-103, miR-107, and miR-122 in chronic EtOH fed rodents [80]. In summary, these data put microRNAs into a crucial position in the regulation of alcoholic liver disease in mice and human, although their role in chronic alholic liver disease is still not fully clarified.

## 5. miRNAs in Liver Fibrosis and Cirrhosis

Liver cirrhosis is representing the common end stage of most chronic liver diseases and it is associated to a tremendous morbidity and mortality. However, treatment options are limited. In this context, miRNAs were suggested as promising new targets for the development of innovative tools for the modulation of this disease. The activation and trans-differentiation of hepatic stellate cells upon exposure of the liver to toxins, or other adverse stimuli, represents a key process in the development of liver fibrosis or cirrhosis [81]. HSCs are the major cell type that is involved in the development of liver fibrosis by secreting ECM molecules like collagens, laminin, proteoglycans and fibronectin. Upon activation, HSC transform into myofibroblast-like cells that produce ECM leading to liver fibrosis [81]. By using array based approaches, it was demonstrated that whole panels of miRNAs are deregulated in activated HSCs, when compared to quiescent HSC. While e.g., miR-29c*, miR-501, miR-349, miR-325-5p, miR-328, miR-143, and miR-193 displayed a significant upregulation, miR-341, miR-20b-3p, miR-15b, miR-16, miR-375, miR-122, miR-146a, miR-92b, and miR-126 were found to be downregulated [82,83,84,85]. The following miRNAs are most noteworthy in the context of liver fibrosis and should be discussed in more details:

**miR-29.** The most widely studied miRNA in liver fibrosis is miR-29 family that is known to have strong anti-fibrotic role in clinical specimen and animal models. The miR-29 family with its members miR-29a, miR-29b, and miR-29c have the same seed sequence AGCACCA. The first instance showing the downregulation of miR-29 in cardiac fibrosis with the altered expression of collagens indicates a therapeutic target for tissue fibrosis [86]. Subsequently, Roderburg and colleagues applied an unbiased miRNA profiling on CCl_4_-treated livers of C57BL/6 mice and detected the downregulation of all three miRNA-29 family members [27]. In regard to HSC, Kupffer cells, hepatocytes, and liver sinusoidal endothelial cells (LSEC), the highest expression of miR-29b was observed in HSC. The function of miR-29 family is linked to various signaling pathways, including nuclear factor 'kappa-light-chain-enhancer' of activated B-cells (NFκb) pathway, TGFβ, and PI3K/AKT signaling for the progression of liver fibrosis [27]. TGFβ stimulated HSC showed a decrease in miR-29 family during hepatic fibrosis by activating AP-1, which is present in the promoter of miR-29 [87,88,89]. Furthermore, Liang et al. showed that miR-29 overexpression suppressed the expression of SMAD3 and TGFβ1, revealing a crosstalk between miR-29b and TGFβ1/Smad3 pathway in promotion of hepatic fibrosis. Moreover, miR-29b prevents liver fibrogenesis by regulating HSC cell proliferation and apoptosis through its binding to the downstream effectors PIK3R1 and AKT3 [29]. Furthermore, downregulation of miR-29 was observed in patients with HCV-induced fibrosis and TaqMan miRNA profiling revealed downregulation of miR-29 and a depressed extracellular matrix synthesis during HSC activation [28]. Beside pro-fibrotic TGFβ signaling, miR-29 is also regulated by pro-inflammatory pathways [27]. Finally, trans-differentiation of HSC into myofibroblasts resulted in the loss of miR-29 expression, an increase of its target genes *PDGF-C,* as well as *IGF-1,* and therefore interfering with profibrogenic cell communication [89]. Furthermore, another interesting study showed that sex hormones play an important role in the development of hepatic fibrosis [90]. Estradiol enhanced miR-29a/b expression via modulation of transcriptional factors NFκb and STAT-1, being in stark comparison to that of TGFβ1. Therefore, miR-29 plays a crucial role in integrating cross-talk between pro-fibrotic and inflammatory signals in HSC, making it a promising anti-fibrotic candidate. 

**miR-34.** The three members of the miR-34 family including miR-34a, miR-34b and miR-34c have differential expression in mouse and human. In mice, miR-34a is highly expressed in the brain, whereas it is highly expression in human in the ovary, prostrate, and testes [91]. Li et al. showed the upregulation of miR-34 family in dimethylnitrosamine-induced hepatic fibrosis in rats [30]. The *acyl-CoA synthetase long-chain family member 1* (ASCL1) is the direct target of miR-34a and miR-34c [30]. Moreover, miR-34a was upregulated in activated HSCs by regulating the deposition of ECM proteins such as collagen, desmin, and αSMA [31]. MiR-34 has pleiotropic roles in cell cycle, apoptosis, and cellular development. Bioinformatic analysis and luciferase reporter assays identified *Peroxisome proliferator-activated receptor gamma* (PPARγ) as a target of miR-34a and miR-34c. Furthermore, *PPARγ* expression was negatively correlated with expression of both miRNAs during HSC activation. In contrast, inhibition of miR-34a and miR-34c in activated HSC led to an upregulation of *PPARγ* expression and reduction of αSMA levels [32]. Yet another study suggests the contribution of miR-34a to alcoholic liver injury as *Caspase-2* and *Sirtuin 1* are direct targets of miR-34a. Moreover, dysregulation of miR-34a led to an altered expression of *matrix metalloproteases* (MMP) *1* and *2* [33]. Finally, miR-34a plays role in regulation of drug- or lipid- metabolizing enzymes. p53 activation gives rise to the expression of miR-34a with a decrease in RXRα protein in fibrotic livers [92].

**miR-122.** miR-122 is one of the most abundant miRNAs in the liver, accounting for 70% of all miRNAs and regulate several functions, including cell cycle, differentiation and apoptosis [93]. The hepatic levels of miR-122 in a cohort of 84 chronic hepatitis C-infected fibrotic patients decreased significantly with the severity of fibrosis [34]. miR-122^−/−^ mice showed inflammation and portal fibrosis due to the activation of HSC [35]. The pro-fibrogenic transcription factor KLF6, representing a direct target of miR-122, was found to be activated in hepatocytes of miR-122 deficient mice. Moreover, despite other data suggested that this might represent an artifact caused by improper cell isolation techniques [94], the group of Li et al. suggested that expression of miR-122 was lower in activated HSCs of mice treated with CCl_4_ regulating collagen production via targeting 3’UTR of P4HA1 mRNA [95]. MiR-122 was markedly decreased in TGFβ -activated primary HSCs through an increase in fibrosis-related genes, including α*-SMA*, *Col1A1*, and *FN1* [24]. Interestingly, the reintroduction of miR-122 in mice with CCl_4_-treated liver fibrosis inhibited the formation of collagen fibrils, suggesting miR-122 to be a promising target for anti-fibrosis therapy. Furthermore, hepatic miR-122 expression levels were upregulated in patients with mild fibrosis than those with severe fibrosis, indicating a useful marker of liver fibrosis in patients with NAFLD [36]. 

## 6. miRNAs in HCC

Hepatocellular carcinoma (HCC) represent the most important primary liver cancer and has risen to become the fifth most common cancer worldwide, with 782,451 known cases in 2012. While improved treatment options have resulted in reduced mortality of distinct malignancies, the incidence of HCC still nearly equals its mortality rate. Even in medically developed countries, patients with advanced HCC face five-year survival rates of less than 10%. [96]. Due to the limited knowledge of its pathophysiology no causative treatment could be established until now. The heterogeneity of HCC represents one of the biggest challenges in dissecting novel targets for a potential use in precision medicine for this disease. This heterogeneity is caused by the varying etiological factors underlying hepatocarcinogenesis, including environmental factors, such as hepatitis B virus (HBV), hepatitis C virus (HCV), alcoholic steatohepatitis, NASH [97], and chemical carcinogens. Genes, reliably been identified in the context of HCC include those regulating cancer related processes such as cell cycle, apoptosis and cell signaling. Increasing attention has been given in the last years to alterations of noncoding RNAs (ncRNAs) expression during HCC development and/ or progression. NcRNAs include (but are not limited to) ribosomal RNAs, small nucleolar RNA, small interfering RNAs, miRNAs, and long non coding RNAs (lncRNAs) [98]. Out of these, most data are available for the specific role of miRNAs in HCC. Out of the many miRNAs that are regulated in HCC development or progression, the liver specific miR-122 and miR-199 are most noteworthy and should be discussed in more details:

**miR-122.** Expression of miRNA-122 has been demonstrated to be downregulated in liver samples from patients with hepatocellular carcinoma, as well as from rodent models of liver cancer [6]. Expression of miR-122 inversely correlated with clinical features, such as development/presence of metastatic disease and the patients´ general prognosis [6]. Moreover, its expression correlated with increased cell proliferation and decreased apoptotic cell death. On a functional level, miR-122 was shown to inhibit proliferation and migration, and to promote hepatocyte deaths in HCC cell lines, as well as in mice [37,38]. In knock-out studies, the deletion of miR-122 was associated with the development of steatohepatitis, fibrosis and in some cases the development of liver cancer. Notably, artificial delivery of miR-122 into miR-122^−/−^ inhibited the formation of HCC, highlighting the role of this miRNA in the pathophysiology of HCC [35,39]. On a molecular level it was demonstrated that miR-122 negatively regulates the expression of *cyclin G1*, leading to elevated *p53*-expression [99]. Moreover, miR-122 regulates *A disintegrin and metalloprotease 10* (ADAM10), *serum response factor* (SRF), and *insulin-like growth factor 1 receptor* (IGF-1R), all promoting hepatocarcinogenesis. Besides these, members of the Wnt/b-catenin signaling pathway and *Pituitary tumor-transforming gene 1* (PTTG1) pathway are targeted by miR-122 and might lead to HCC development in case of mIr-122 down-regulation [37,100,101].

**miR-199 family.** Downregulation of miR-199a-5p was recognized as a frequent event in HCC and down-regulation of miR-199a-5p were associated to more advanced disease stages. Moreover, down-regulation of this miRNA was associated with higher recurrence rates after tumour resection and an impaired patients’ prognosis [40]. In HCC cell lines, miR-199a was found to suppress tumor proliferation and to induce apoptosis and cell cycle arrest by regulating the expression of *matrix metallo-proteinase-9* (MMP-9), *frizzled type 7 receptor* (FZD7), and *hypoxia-inducible factor-1α* (HIF1α) [41,42,43]. Notably artificial overexpression of miR-199a-3p prevented development of hepatocellular carcinoma in vitro. Functionally, the increased miR-199a-3p levels remarkably suppressed cell proliferation and migration, as well as induced cellular apoptosis. Other targets of miR-199a include the tumor-promoting *p21* (*RAC1*) *activated kinase 4* (PAK4), the *mammalian target of rapamycin* (mTOR) and *c-Met*, which have been described as tumor suppressors in the context of HCC [102,103,104,105]. Other studies demonstrated that miR-199a suppresses tumor growth in hepatocellular carcinoma by targeting pro-angiogenic mediators [106], highlighting the pleotropic function that miR-199s plays in the context of HCC and makes this miRNA family to an attractive target when considering target miRNAs for HCC treatment [104].

## 7. Outlook

Since the discovery of the first microRNA (miRNA), tremendous efforts have been made in the field of miRNA biology. Based on the findings on the role of miRNAs in cancer development and progression, miRNAs are regarded as attractive tools and targets for new therapeutic approaches. Several studies have used animal models to demonstrate that liver fibrosis might be ameliorated or even reversed by treatment with agomirs or antagomirs. As an example, LNA-anti-miR-214 had a preventive effect for hepatic fibrosis, while inhibition of miR-21 reduced liver fibrosis through reduction of CD24^+^ liver progenitor cells. In this context, Miravirsen, a locked nucleic acid-modified DNA phosphorothioate antisense oligonucleotide that inhibits miR-122, and that was tested in a phase 2a trial in patients with viral hepatitis, lead to a dose-dependent reduction in HCV RNA levels, highlighting the feasibility of miRNA-based therapies in human. In a recent phase 1 trial MRX34, a liposome-formulated mimic of the tumor suppressor miRNA miR-34, showed antitumor activity in patients with refractory advanced solid tumors. Based on these results, more large randomized prospective clinical trials are currently planned to further test the application of miRNAs in the context of liver diseases. 

## 8. Conclusions

miRNAs play a crucial role in the pathophysiology of liver toxicity and liver injury. miRNAs represent promising targets for the development of strategies to identify, prevent, or treat these disease states. Moreover, circulating miRNAs reflect pathological conditions of the liver and might be used as non-invasive markers for the diagnosis liver pathologies.

## Figures and Tables

**Table 1:** Representative overview on miRNAs involved in acute in chronic liver diseases.

miRNA	Model/Side of Action	Findings	Reference
**Acute Liver Injury**			
miR-122	APAP mice model	elevated serum levels, Elevation dose and exposure dependent	[15]
	I/R mice model	elevated miR-122 serum levels, correlation with AST, ALT and hepatic cell death	[16]
	APAP induced ALF patients	elevated serum levels	[17,18]
	*Dioscorea bulbifera* induced liver injury	elevated serum levels	[19]
miR-223	*miR-223^−/−^* mice in FAS induced liver injury model	protection against hepatocyte apoptosis and liver injury	[20]
	APAP mice model	upregulation of miR-223	[21]
	ConA mice model	upregulation of miR-223	[21]
	acute CCl_4_ and I/R mice model	upregulation; KO had no effect on severity of liver damage;	[21]
	ALF patients	elevated liver tissue and serum levels; Impaired prognosis for patients with elevated miR-223 tissue levels; identification of miR-223 as potential biomarker for liver damage	[21]
miR-150	*miR-150^−/−^* in FAS induced liver injury model	miR-150 deficiency had protective effect; elevated *AKT* expression *AKT1* and *AKT2* are direct targets of miR-150	[22]
	*miR-150^−/−^* in LPS/GLN	no effect observed	[22]
miR-192-5p	HepG2 cells treated with H_2_O_2_	identification of Zeb2 as miRNA target regulating cell death	[23]
	I/R and CCl_4_ mice model/ALF patients	downregulated in liver; serum levels increased after I/R and correlated with degree of liver damage	[24]
miR-1224	I/R, APAP and CCl_4_ mice model	upregulation was associated with impaired proliferation and elevated apoptosis In hepatocytes: miR-1224 repressed the anti-apoptotic gene *Nfib*	[25]
	ALF patients	elevated serum and liver tissue levels were linked to unfavourable prognosis	[25]
**Chronic Liver Diseases**			
miR-155	*miR-155^−/−^* in alcohol mice model	KO protected from alcohol induced steatosis, inflammation and liver fibrosis	[26]
miR-29	CCl_4_ mice model	downregulation of miR-29a, miR-29b and miR-29c;	[27]
	IFNα & TGFβ1 stimulated HSC,	decreased miR-29 expression; reduced ECM synthesis	[28]
	Ectopic expression of miR-29b in activated HSCs (LX-1, HSC-T6)	miR-29b suppressed *SMAD3* and *TGFβ1* and prevents liver fibrosis by regulating HSC proliferation and apoptosis through its targets *PIK3R1* and *AKT3*	[29]
miR-34	Dimethylnitrosamine-induced hepatic fibrosis in rats	upregulation of miR-34 family; ASCL1 is a direct target of miR-34a and miR-34c	[30]
	activated HSC	upregulation of miR-34a was associated with regulation of ECM proteins like collagen, desmin a αSMA Identification of *PPARγ* as target of miR-34a and miR-34c	[31,32]
	alcoholic liver injury model	*Caspase 2* and *Sirtuin 1* are direct targets of miR-34; *MMP1* and *MMP2* were dysregulated after altered miR-34a expression	[33]
miR-122	chronic hepatitis C patients	decreased hepatic expression miR-122 correlated with severity of fibrosis	[34]
	*miR-122^−/−^*	displayed inflammation and portal fibrosis due to activation of HSC; pro-fibrotic transcription factor KLF6 is a direct target of miR-122 and was activated in hepatocytes of miR-122^−/−^ mice.	[35]
	Reintroduction of miR-122 in CCl_4_ treated mice	Inhibition of Collagen fibrils formation	
	NAFLD patients	high miR-122 expression was associated with more severe liver fibrosis	[36]
**Hepatocellular Carcinoma**			
miR-122	HCC patients	downregulated miR-122 expression in HCC patients. Expression inversely correlated with presence of metastatic disease and patients´ general prognosis	[6]
	HCC cell lines	miR-122 inhibit proliferation, migration and promotes hepatocyte death	[37,38]
	*miR-122^−/−^*	miR-122 deletion was associated with development of steatohepatitis, fibrosis and liver cancer	[35,39]
miR-199	HCC patients	downregulation of miR-199a-5p in HCC was associated with more advanced disease stages, higher recurrence rates and impaired overall patients’ prognosis	[40]
	HCC cell lines	miR-199a suppressed tumour proliferation, induced apoptosis and cell cycle arrest (via regulation of *MMP-9, FZD7, HIF1α*)	[41,42,43]

Abbreviations: AKT1, AKT serine/threonine kinase 1; AKT2, AKT serine/threonine kinase 2; ALF, acute liver failure; ALT, alanine transaminase; APAP, acetaminophen; AST, aspartate aminotransferase; CCl_4_, carbontetrachloride; ConA, concanavalin A; ECM, extracellular matrix; FAS, Fas cell surface death receptor; HCC, hepatocellular carcinoma; HSC, hepatic stellate cells; INFα, interferon α; I/R, ischemia and reperfusion; KO, knock-out; miRNA, micro-RNA; MMP, matrix metalloproteases; PIK3R1, phosphoinositide-3-kinase regulatory subunit 1; SMAD3, SMAD family member 3.

## Abbreviations

ADAM10	A disintegrin and metalloprotease 10
ALF	Acute liver failure
ALT	Alanine transaminase
APAP	Acetaminophen
AST	Aspartate aminotransferase
ASCL1	Acyl-CoA synthetase long-chain family member 1
CCl_4_	Carbontetrachloride
ConA	Concanavalin A
d-Gal	d-Galactosamine
ECM	Extracellular matrix
FZD7	Frizzled type 7 receptor
HBV	Hepatitis B virus
HCC	Hepatocellular carcinoma
HCV	Hepatitis C Virus
HIF1α	Hypoxia-inducible factor-1α
IGF-1R	Insulin-like growth factor 1 receptor
I/R	Ischemia and reperfusion
lncRNAs	Long non coding RNAs
LPS	Lipopolysaccharide
LSEC	Liver sinusoidal endothelial cells
miR	Micro-RNA
MMP	Matrix metalloproteases
mTOR	Mammalian target of rapamycin
NAFLD	Non-alcoholic fatty liver disease
NASH	Non-alcoholic steatohepatitis
ncRNA	Noncoding RNAs
NFκb	Nuclear factor ‘kappa-light-chain-enhancer’ of activated B-cells
PAK4	P21 (RAC1) activated kinase 4
PPARγ	Peroxisome proliferator-activated receptor gamma
PTTG1	Pituitary tumor-transforming gene 1
SRF	Serum response factor
TAA	Thioacetamide
